# Spontaneous Aortic Haematoma in a Patient Receiving Adjuvant Folinic Acid, 5-Fluorouracil, Irinotecan, and Oxaliplatin Chemotherapy Following Resection of a Pancreatic Adenocarcinoma

**DOI:** 10.7759/cureus.10908

**Published:** 2020-10-12

**Authors:** Ruth Dacie, Laura Woodhouse, Damian Mullan, Angela Lamarca

**Affiliations:** 1 Medical Oncology, The Christie NHS Foundation Trust, Manchester, GBR; 2 Radiology and Interventional Radiology, The Christie NHS Foundation Trust, Manchester, GBR; 3 Division of Cancer Sciences, The University of Manchester, Manchester, GBR

**Keywords:** aortitis, aortic haematoma, vasculitis, vascular toxicity, chemotherapy, pancreatic adenocarcinoma, drug-induced

## Abstract

This case report describes the finding of a spontaneous aortic haematoma in a patient receiving adjuvant folinic acid, 5-fluorouracil, irinotecan, and oxaliplatin (FOLFIRINOX) chemotherapy following resection of a pancreatic adenocarcinoma. The haematoma was thought to have arisen secondary to a chemotherapy-induced vasculitis affecting the aorta, as the patient had no other risk factors for de novo aortitis or aortic haematoma. There have been several previously documented cases of associations between chemotherapy agents (in particular platinum-based agents) and vasculitis. This report includes computed tomography (CT) images and a discussion of the literature of related cases. This case is a good example of rare vascular toxicities arising from chemotherapy, the impact such rare complications have upon further chemotherapy options, and how these should be discussed when consenting patients for chemotherapy due to the potentially life-threatening complications.

## Introduction

Pancreatic adenocarcinoma is a major cause of cancer-related morbidity and mortality in the developed world [1]. In localized pancreatic carcinoma, surgical resection offers a chance of cure, though five-year survival rates after surgical resection alone are low (10-20%) [2-4]. Adjuvant chemotherapy following resection has been shown to improve outcomes, although recurrence is common and prognosis remains poor [1,5].

Folinic acid, 5-fluorouracil, irinotecan, and oxaliplatin (FOLFIRINOX) chemotherapy is a common chemotherapy regime used in pancreatic adenocarcinoma. FOLFIRINOX was first reported as a therapy for pancreatic carcinoma in 2010. The ACCORD-11 trial found that it produced a survival benefit compared to single-agent gemcitabine, the previous standard of care, in patients with metastatic pancreatic cancer and good performance status [6]. Subsequently, another large trial found that FOLFIRINOX was also superior to gemcitabine in the adjuvant setting for patients with resected pancreatic adenocarcinoma [7]. This study included patients with both R0 (no cancer cells within 1mm of all resection margins) and R1 (cancer cells present within 1mm of one or more resection margins) resections.

Compared to regimes with fewer chemotherapy agents, FOLFIRINOX has relatively high toxicity rates; common toxicities include neutropenia, febrile neutropenia, thrombocytopenia, diarrhea, and sensory neuropathy [6,7]. As a result, it is common for patients to receive modifications to their treatment regimes, most commonly dose reductions or deferrals of treatment cycles.

## Case presentation

A 58-year-old female patient was diagnosed with localised pancreatic adenocarcinoma in December 2019. She had a past medical history of hypertension and hypothyroidism. Her only ongoing medication was levothyroxine; she had recently stopped ramipril due to hypotension. Following a Whipple resection (pancreaticoduodenectomy) in January 2020, the diagnosis of pancreatic adenocarcinoma with pathological stage of pT2N2 was confirmed; resection margins were positive (R1). Adjuvant chemotherapy with modified FOLFIRINOX chemotherapy (two-week cycles of folinic acid, 5-fluorouracil, irinotecan, and oxaliplatin) was commenced in March 2020. Following cycle two, she had a 10-day admission with neutropenic colitis. Bloods taken prior to cycle three showed an acute kidney injury (AKI); due to this, cycle three was deferred by a week and a 25% dose reduction to all chemotherapy drugs was applied.

Two weeks after the third cycle of chemotherapy, she was admitted with a fever and episodes of back pain. She described episodes of sharp pain in the mid-thoracic region of her back, sometimes precipitated by eating. Of note, she had recently completed a seven-day course of oral amoxicillin/clavulanic acid for a *Klebsiella pneumoniae* urinary tract infection, but had, at the time of this admission, no symptoms of urinary tract infection. The patient also described a transient maculopapular rash affecting her upper arms, which had been present a week earlier, but had resolved by the time of admission. On admission, she was commenced on broad-spectrum intravenous antibiotics (piperacillin/tazobactam) and a septic screen was performed.

**Figure 1:**
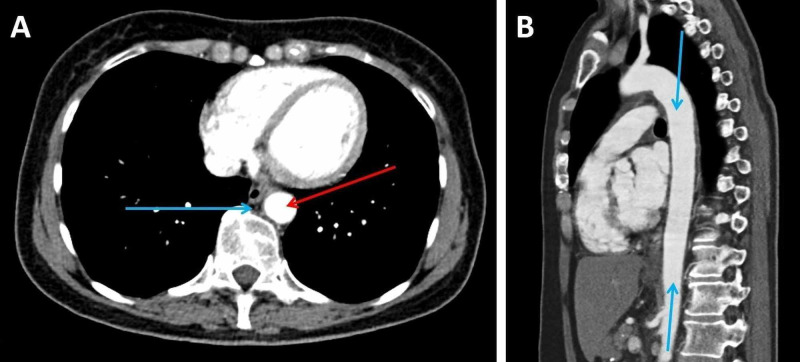
CT imaging of the thorax, abdomen and pelvis performed prior to commencement of chemotherapy (A: transverse plane; B: sagittal plane) A: This image shows a normal posterior mediastinum. The aorta (red arrow) closely abuts the vertebra. The lumen is normal and measures 19mm x 20mm in diameter. The mediastinal fat is barely perceptible and appears normal (blue arrow). B: This image shows a normal calibre thoracic aorta (blue arrows).

**Figure 2:**
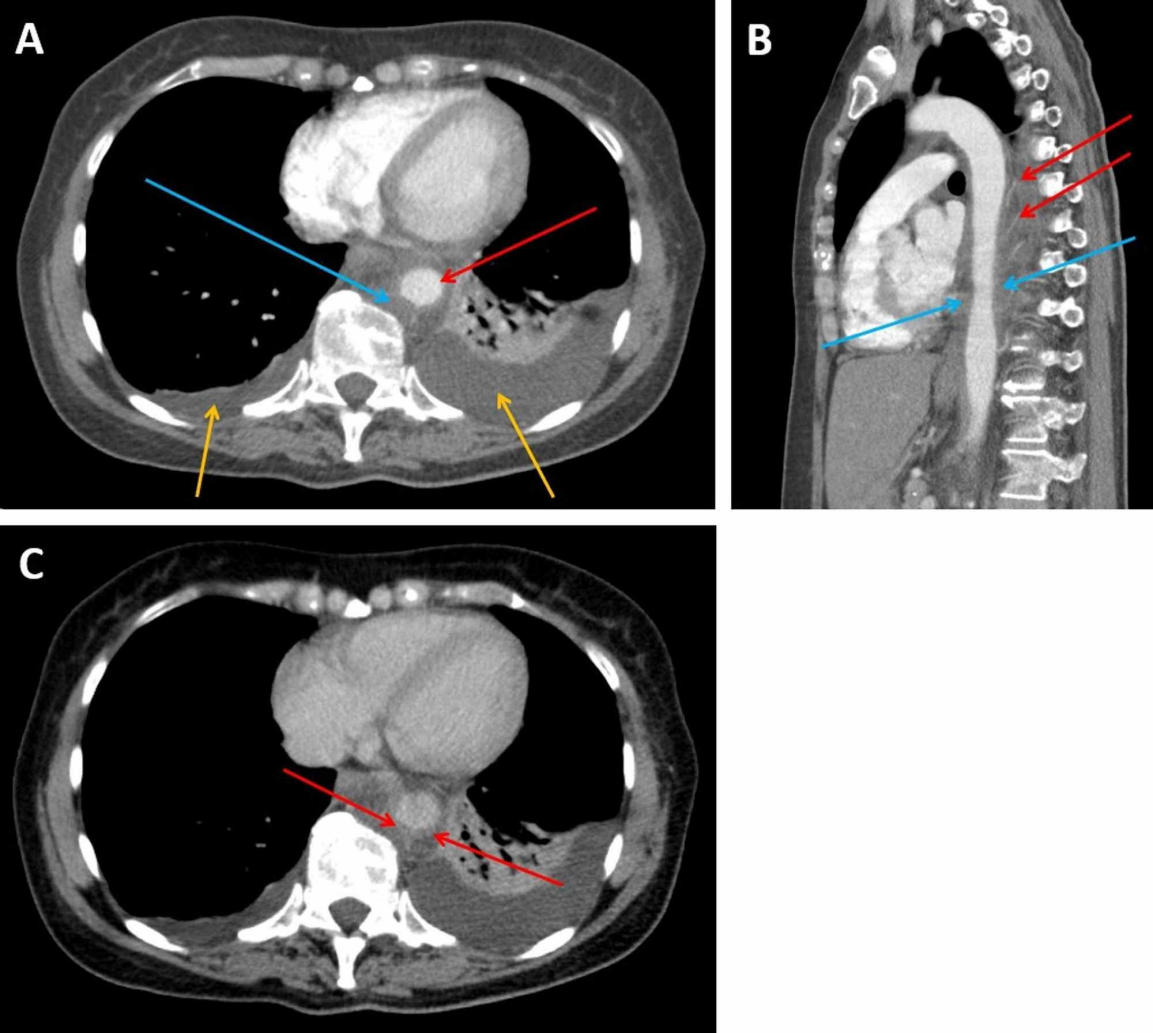
CT imaging of the thorax, abdomen and pelvis performed on the fourth day of admission (A: transverse plane, arterial phase; B: sagittal plane, arterial phase; C: transverse plane, portal venous phase) A: This arterial phase image shows expansion and opacification of the posterior mediastinal fat due to haemorrhage. The aorta (red arrow) has been displaced from the vertebra and is compressed by a peri-aortic haematoma (blue arrow). The lumen is now narrowed measuring 18mm x 16mm. Bilateral pleural effusions are also present (yellow arrows). B: This arterial phase image demonstrates ectatic narrowing of the aorta (blue arrows) with circumferential hyperdensity indicating peri-aortic haematoma and splaying of the intercostal arteries reflecting mass effect (red arrows). C: This image, taken in the portal venous phase, better demonstrates the aortic mural thickening (red arrows).

Blood culture samples were obtained peripherally and centrally from the indwelling tunneled intravascular access device (port-a-cath). These showed no bacterial growth. Urine cultures were negative, nose and throat swabs for severe acute respiratory syndrome coronavirus 2 (SARS-CoV-2) and serum beta-D-glucan were also negative (<31pg/ml). A chest x-ray was performed and found to be normal, with no lung or mediastinal abnormality detectable. Serial blood tests revealed a drop in haemoglobin (from 103g/L two days prior to admission to 76g/L on the second day of admission (normal range 115-165g/L) and the patient continued to spike temperatures >38°C. A computed tomography (CT) scan of the thorax, abdomen, and pelvis was performed to investigate the source of the patient’s fever, back pain, and anaemia. The images were compared to the baseline CT performed prior to starting chemotherapy (Figure 1). The CT demonstrated high-attenuation fat stranding around the descending thoracic aorta, extending from the level of the carina to the diaphragmatic crura. There was some mass effect with compression and displacement of the aorta (Figure 2). This was presumed to represent a mediastinal haematoma secondary to aortitis, with no evidence of active bleeding. Also noted were bilateral pleural effusions and stable soft tissue thickening around the superior mesenteric vessels, taken to be post-surgical. There was no evidence of cancer recurrence.

Following discussion with cardiothoracic and vascular surgical teams, a dedicated CT angiogram of the aorta was performed, with unenhanced, arterial, and portal venous phase imaging. In addition to the aortic compression and peri-aortic haematoma, this revealed mild thickening of the media and adventitia of the distal thoracic aorta, further confirming a diagnosis of aortitis. Branches of the aorta (including renal arteries) were unremarkable. Further investigations included a transthoracic echocardiogram, which showed no aortic root pathology, no valvular pathology, and normal systolic function.

In summary, the initial working diagnosis was that of aortitis, with an acute intramural haematoma of the descending aorta and peri-aortic extension. It was thought possibly to have occurred de novo, although a background drug-induced aortitis was also considered possible until further investigations for vasculitis could be performed. Vasculitis screening tests, including anti-neutrophil cytoplasmic antibody (ANCA), anti-double-stranded DNA (anti-dsDNA), anti-centromere antibody, serum immunoglobulins, and paraprotein and syphilis serology were subsequently performed; all of which were found to be within normal limits. Both C-reactive protein (CRP) and erythrocyte sedimentation rate (ESR) were elevated; highest CRP was 210mg/L on the fourth day of admission (normal range 0-5mg/L) and highest ESR was 120mm on the sixth day of admission (normal range 0-22mm).

The patient’s blood pressure was monitored; on the day of admission and throughout the admission blood pressure was predominantly between 130-140 systolic and 60-70 diastolic (range 104/60 to 177/92). Amlodipine was initiated to achieve a target blood pressure of <130/80. The presence of haemothorax was excluded by diagnostic sampling of the pleural effusions; the fluid was serous with normal biochemistry. Cytology analysis revealed the presence of benign mesothelial cells and leucocytes, but no malignant cells.

The CT angiogram was repeated eight days following the initial scan; this showed improvement in the aortic and peri-aortic inflammation and reduction in size of the pleural effusions. The patient received 10 days of intravenous antibiotics (piperacillin/tazobactam then meropenem) followed by seven days of oral antibiotics (amoxicillin/clavulanic acid). During this time, her symptoms resolved and her inflammatory markers improved; CRP fell to 25mg/L. She was discharged home after 14 days as an inpatient.

**Figure 3:**
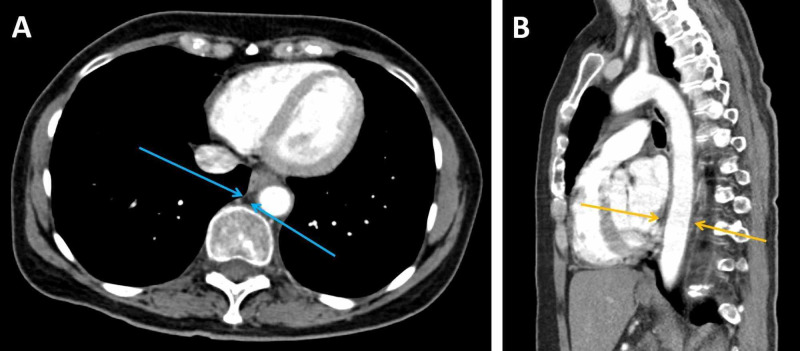
Follow-up CT imaging of the thorax, abdomen and pelvis performed six weeks after discharge (A: transverse plane, arterial phase; B: sagittal plane, arterial phase) A: This image shows resolution of the peri-aortic haematoma and restoration of the normal mediastinal fat in the posterior mediastinum (blue arrows) B: This image shows a normal calibre aorta after resolution of the compressive haematoma (yellow arrows)

Whilst the diagnosis of vasculitis was not confirmed histologically, the imaging features were pathognomonic, and aortitis with mediastinal extension as an effect of systemic vascular-damaging chemotherapy (i.e. a drug-induced vasculitis) was felt to be the most likely diagnosis, considering the negative vasculitis screening tests. On this basis, adjuvant chemotherapy was stopped to avoid unnecessary risk and potential further life-threatening complications. A follow-up CT angiogram performed six weeks after discharge demonstrated resolution of the peri-aortic haematoma with restoration of normal appearances of the aorta and mediastinum (Figure 3).

## Discussion

The diagnosis favoured by the vascular multidisciplinary team (MDT) was aortitis with an aortic intramural haematoma; arising either de novo or secondary to a drug-induced vasculitis. In cases of spontaneous aortic intramural haematoma, the precipitating factor is not always identified. Hypertension and atherosclerosis are known to play a major role in most cases, while a small proportion result from trauma [8]. This patient had no history of trauma, but had a history of mild hypertension. She had actually stopped ramipril in recent months due to hypotension. She had no other cardiovascular risk factors or family history. On admission and throughout her hospital admission her blood pressure was well controlled, making a spontaneous haematoma due to hypertension unlikely.

An alternative explanation for the development of the intramural haematoma was a preceding vasculitic process affecting the aorta. Causes of aortitis include autoimmune conditions, infectious agents (in particular syphilis, tuberculosis, HIV, and salmonella), and connective tissue disorders [9]. Paraneoplastic vasculitides have also been described, however 90% of these occur with haematological rather than solid organ malignancies [10].

The normal panel of autoantibodies makes an autoimmune vasculitis unlikely. Apart from a transient maculopapular rash affecting her upper arms, the patient had no other symptoms or signs of vasculitis. The fact that the patient’s fever and inflammatory markers seemed to respond to antibiotic therapy makes an infectious cause for her presentation a possibility, however one would expect positive blood cultures for an infective aortitis, and no specific pathogens were identified.

A chemotherapy-related aortitis remains the most likely explanation, given the lack of other positive findings and risk factors. There are several case reports describing associations between vasculitis and platinum-based chemotherapy agents; one reports a case of aortitis during cisplatin-based chemotherapy [11]; another describes two cases of leukocytoclastic (small-vessel) vasculitis during oxaliplatin-based chemotherapy [12]. In all of these cases, levels of autoantibodies and immunoglobulins were within the normal ranges.

Associations between non-platinum-based chemotherapy agents and vasculitis have also been proposed. A case of small-vessel vasculitis in a patient receiving combination treatment with 5-fluorouracil, folinic acid, and oxaliplatin has been described [13]; in this case, it could not be determined which of the chemotherapy agents was the most likely cause of the vasculitis. In a separate report, a patient receiving adjuvant chemotherapy with 5-fluorouracil, folinic acid, and oxaliplatin developed small bowel ischaemia secondary to a vasculitic process in the superior mesenteric artery, which was attributed to the 5-fluorouracil [14]. Other vascular toxicities, in particular cardiotoxicity, are well known with 5-fluorouracil, however no cases of aortitis have been described with 5-fluorouracil to date.

In this case, either oxaliplatin or 5-fluorouracil were thought most likely to have caused the aortitis and aortic intramural haematoma. In this adjuvant setting, with a post-operative patient with no recurrent or residual disease identifiable on imaging, a decision was made to cease chemotherapy to avoid unnecessary risk and potential further life-threatening complications.

## Conclusions

Vascular toxicities from chemotherapy are rare, but can arise during treatment. These should be discussed when consenting patients for chemotherapy due to the potential life-threatening complications, and the implications of having to halt chemotherapy. This is particularly pertinent in a disease group such as pancreatic adenocarcinoma, which has low survival rates, high recurrence rates, and few other chemotherapy treatment options.
